# Supplementary figures and images for: Oligogalacturonides Enhance Resistance against Aphids through Pattern-Triggered Immunity and Activation of Salicylic Acid Signaling
Source: Int J Mol Sci. 2022 Aug 28;23(17):9753. doi: 10.3390/ijms23179753 (PMC9456349; doi:10.3390/ijms23179753)

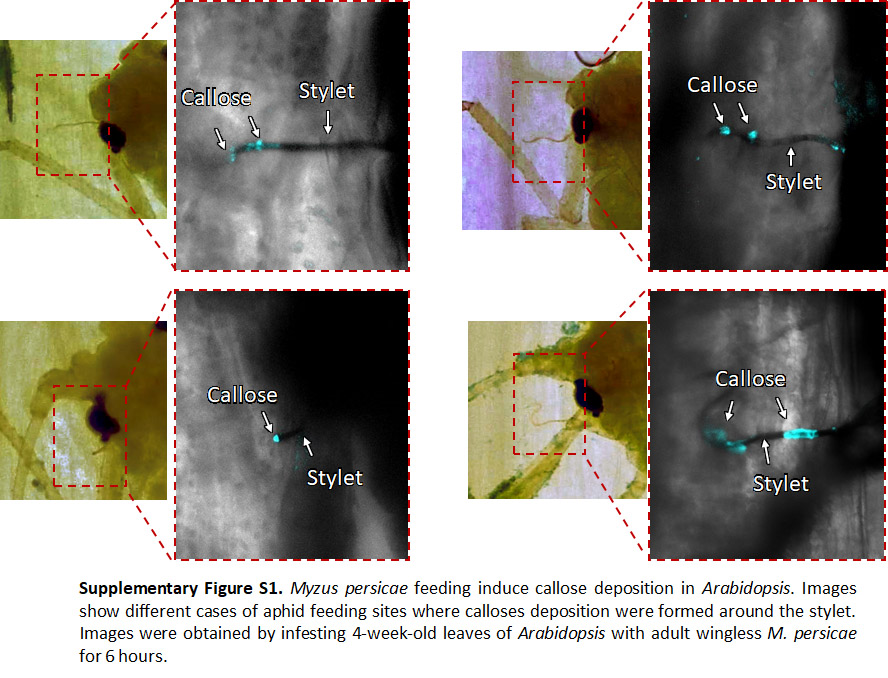

Supplement: Supplementary file 1 [file ijms-23-09753-s001.zip › Supplementary figure S1.jpg]

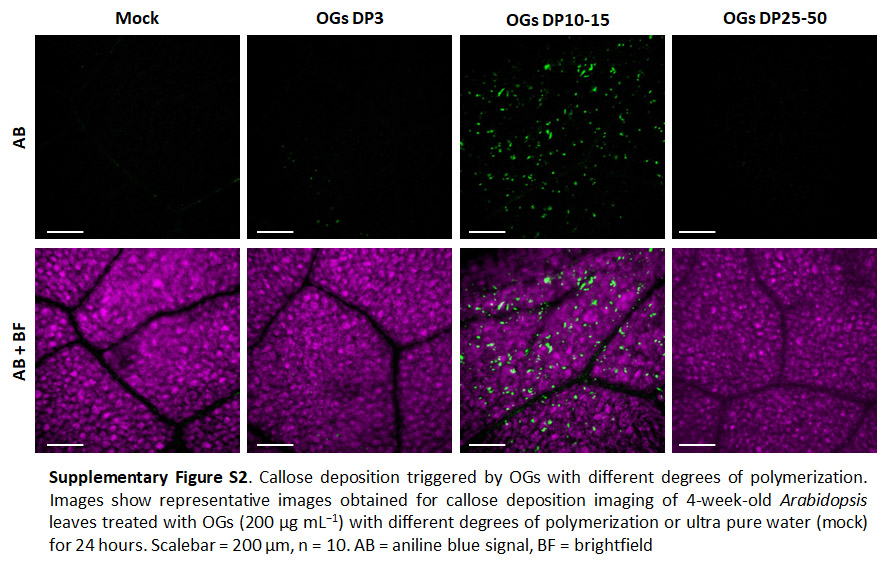

Supplement: Supplementary file 1 [file ijms-23-09753-s001.zip › Supplementary figure S2.jpg]

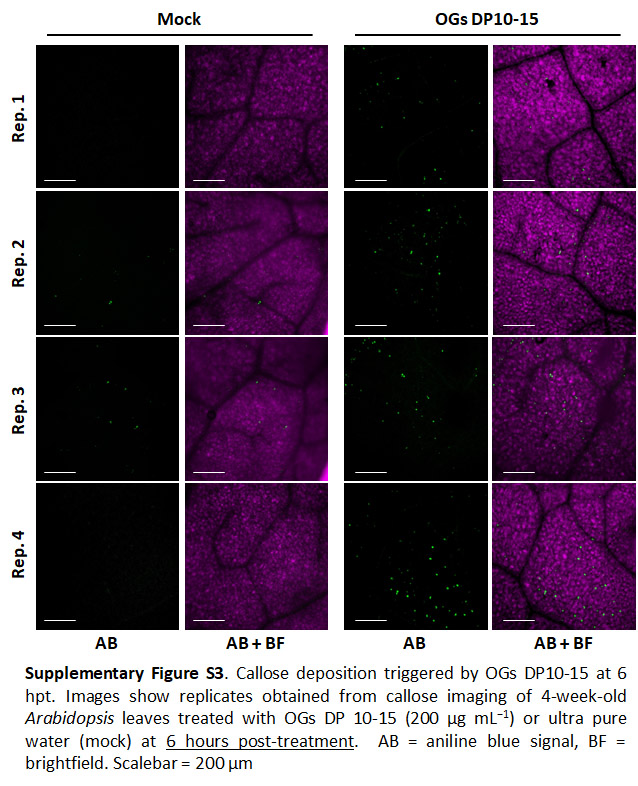

Supplement: Supplementary file 1 [file ijms-23-09753-s001.zip › Supplementary figure S3.jpg]

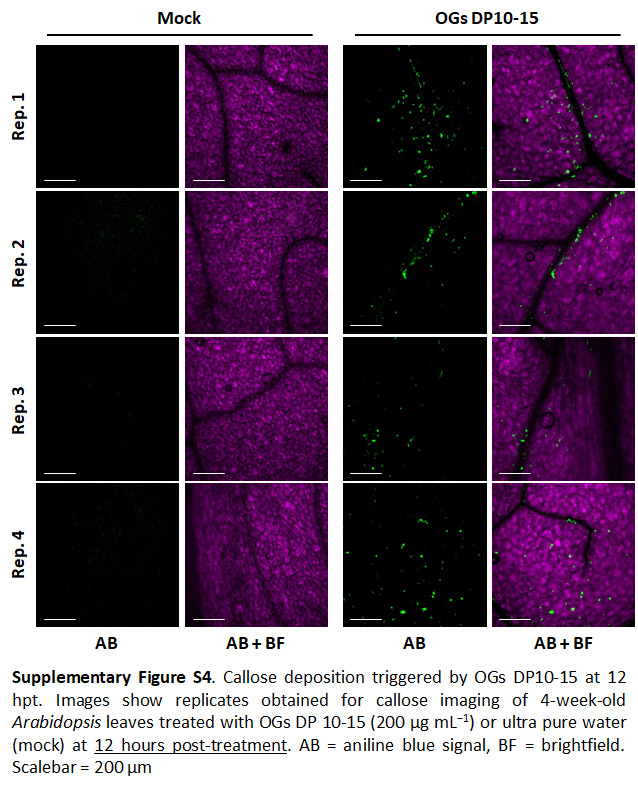

Supplement: Supplementary file 1 [file ijms-23-09753-s001.zip › Supplementary figure S4.jpg]

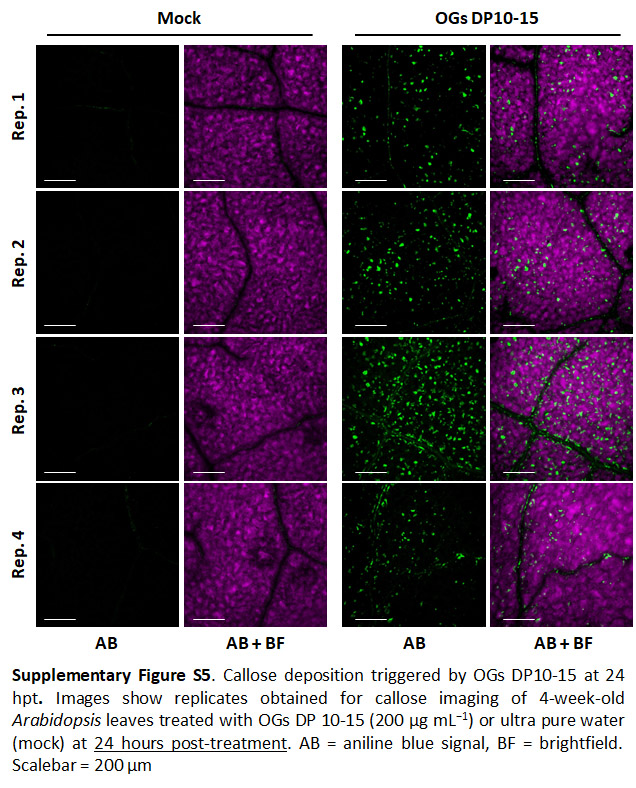

Supplement: Supplementary file 1 [file ijms-23-09753-s001.zip › Supplementary figure S5.jpg]

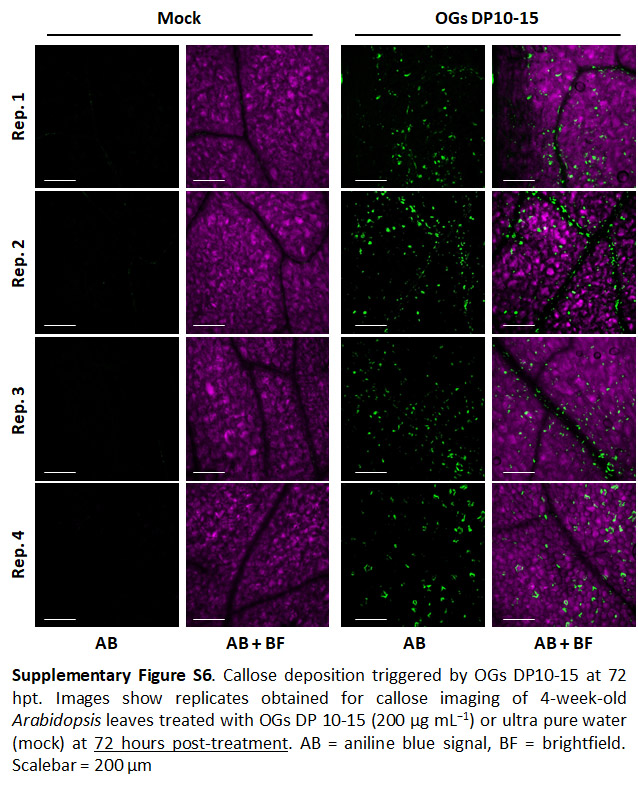

Supplement: Supplementary file 1 [file ijms-23-09753-s001.zip › Supplementary figure S6.jpg]

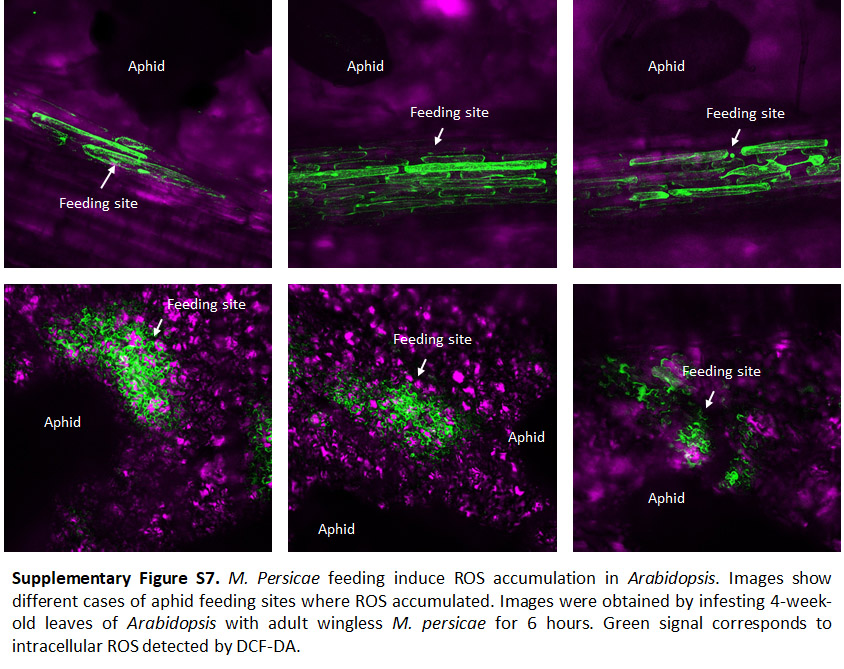

Supplement: Supplementary file 1 [file ijms-23-09753-s001.zip › Supplementary figure S7.jpg]

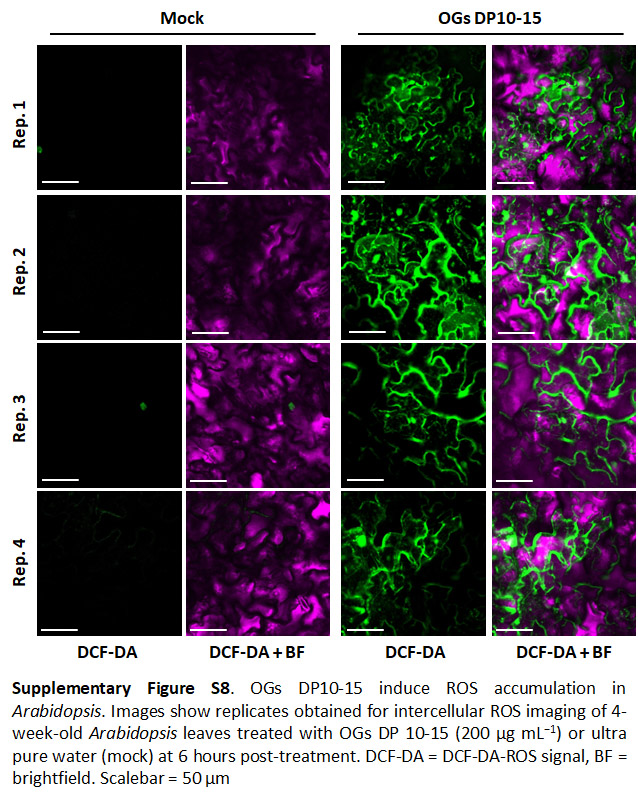

Supplement: Supplementary file 1 [file ijms-23-09753-s001.zip › Supplementary figure S8.jpg]

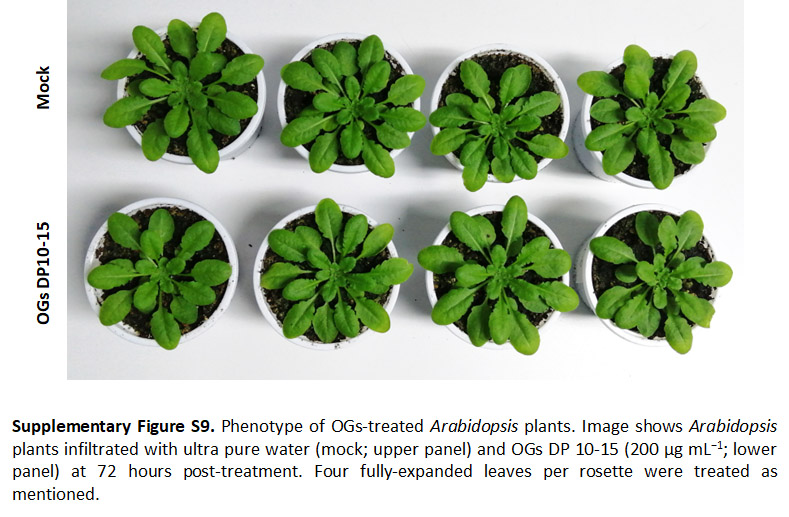

Supplement: Supplementary file 1 [file ijms-23-09753-s001.zip › Supplementary figure S9.jpg]

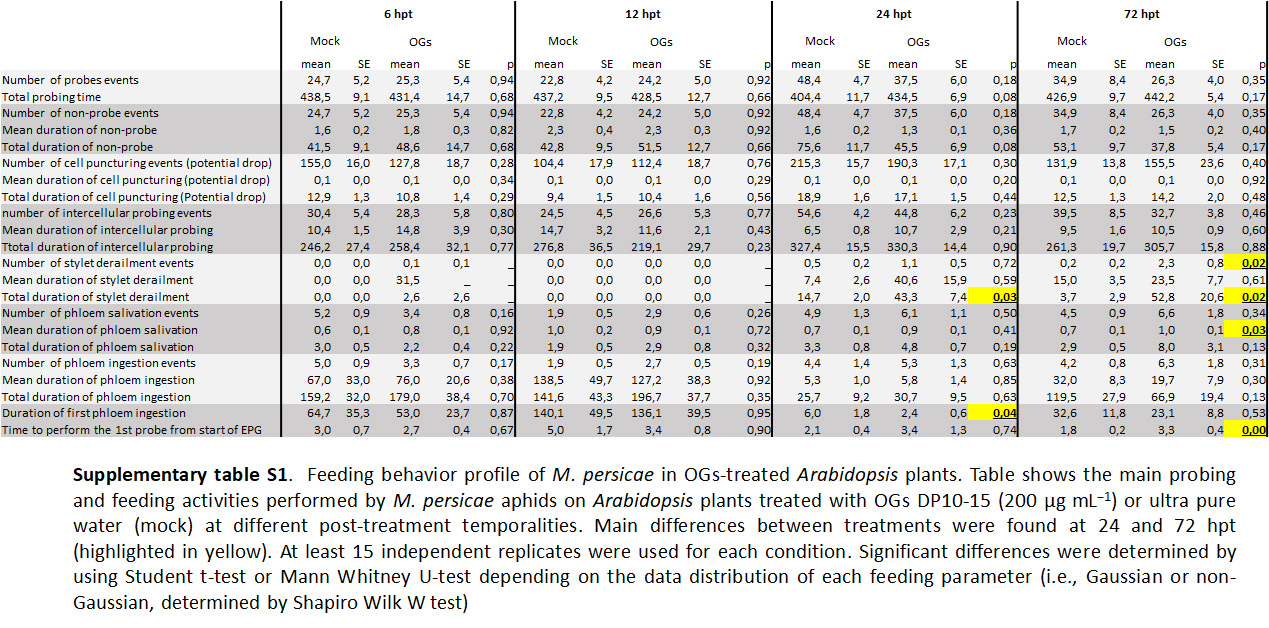

Supplement: Supplementary file 1 [file ijms-23-09753-s001.zip › Supplementary table S1.jpg]
